# Cellular distribution and cytotoxicity of graphene quantum dots with different functional groups

**DOI:** 10.1186/1556-276X-9-108

**Published:** 2014-03-06

**Authors:** Xiaochan Yuan, Zhiming Liu, Zhouyi Guo, Yanhong Ji, Mei Jin, Xinpeng Wang

**Affiliations:** 1 MOE Key Laboratory of Laser Life Science and Institute of Laser Life Science, College of Biophotonics, South China Normal University, Guangzhou 510631, China

**Keywords:** Graphene quantum dots, Chemical modification, Cells, Intracellular distribution, Cytotoxicity

## Abstract

Graphene quantum dots (GQDs) have been developed as promising optical probes for bioimaging due to their excellent photoluminescent properties. Additionally, the fluorescence spectrum and quantum yield of GQDs are highly dependent on the surface functional groups on the carbon sheets. However, the distribution and cytotoxicity of GQDs functionalized with different chemical groups have not been specifically investigated. Herein, the cytotoxicity of three kinds of GQDs with different modified groups (NH_2_, COOH, and CO-N (CH_3_)_2_, respectively) in human A549 lung carcinoma cells and human neural glioma C6 cells was investigated using thiazoyl blue colorimetric (MTT) assay and trypan blue assay. The cellular apoptosis or necrosis was then evaluated by flow cytometry analysis. It was demonstrated that the three modified GQDs showed good biocompatibility even when the concentration reached 200 μg/mL. The Raman spectra of cells treated with GQDs with different functional groups also showed no distinct changes, affording molecular level evidence for the biocompatibility of the three kinds of GQDs. The cellular distribution of the three modified GQDs was observed using a fluorescence microscope. The data revealed that GQDs randomly dispersed in the cytoplasm but not diffused into nucleus. Therefore, GQDs with different functional groups have low cytotoxicity and excellent biocompatibility regardless of chemical modification, offering good prospects for bioimaging and other biomedical applications.

## Background

Quantum dots have been widely applied in the biomedical field due to their various advantages such as size-dependent optical properties, high fluorescence quantum yields, and excellent stability against photobleaching [1-3]. However, the biomedical applications of conventional semiconductor quantum dots which generally composed of the elements from the II-VI group or III-V group (e.g., CdSe) have been greatly limited by the release of heavy metals [1-5]. Recently, carbon luminescent nanomaterials have incited great research interest because of their lower toxicity than semiconductor quantum dots and high photostability compared to organic dyes [6-9].

Graphene is a kind of two dimensional honeycomb structure composed by single layer of sp^2^ carbon atoms, which has been studied in various fields such as optoelectronic devices, energy storage media and drug delivery vectors [10-12]. Graphene quantum dots (GQDs), a kind of zero-dimensional material, have the same single-atom layer as graphene but their lateral dimensions are less than 100 nm [13-16]. Owing to their high surface area and good biocompatibility, GQDs have the potential to be vectors for delivery protein or drug molecules to cells [6,12,17-19]. GQDs can also serve as good fluorescent probes for bioimaging due to their excellent luminescent properties [6,20,21]. Beyond that, when functionalized with different chemical groups, GQDs can be used to build multifunctional structure through combining with various other materials such as protein, drug molecules, and nanotubes by covalent linkage, which will extend their widespread applications in biomedical field [18,22,23]. Jing and his colleagues have fabricated multifunctional core-shell structure capsules composed of olive oil, dual-layer porous TiO_2_ shell, Fe_3_O_4_, and GQDs [23]. In addition, the fluorescence spectrum and the quantum yield (QY) of GQDs also vary with the surface chemical groups on them. Shen and his colleagues have prepared GQDs-PEG with QY as high as 28.0%, which was two times higher than the GQDs (13.1%) without chemical modification [8,24]. Recently, GQDs with different functional groups have excited extensive and increasing research interest.

Up to now, little effort has been focused on the cytotoxicity and distribution research of GQDs with different functional groups. Wu and his colleagues explored the intracellular distribution and cytotoxicity of GQDs prepared through photo-Fenton reaction of graphene oxide (GO) [25,26]. The results demonstrated that this kind of GQDs distributed in the cytoplasm, and their cytotoxicity was lower than that of the micrometer-sized GO [26]. Markovic et al. discovered that electrochemically produced GQDs can be used for photodynamic therapy by inducting oxidative stress and activating both apoptosis and autophagy when irradiated with blue light, which raised a concern about their potential toxicity [27]. Zhu and his colleagues reported that the GQDs that they synthesized did not weaken the cell viability significantly [21]. However, the study from Zhang et al. reported that GQDs synthesized by electrochemical means can be used for efficient stem cell labeling with little cytotoxicity, and they dispersed in the cytoplasm [20]. Some of these results were contradictory, and for the newly developed graphene quantum dots and their derivatives, such information was generally lacking.

In this work, we compared the cytotoxicity of three GQDs with different functional groups (NH_2_, COOH, and CO-N (CH_3_)_2_, respectively) and observed their cellular distribution in human A549 lung carcinoma cells and human neural glioma C6 cells. The acquired results will provide valuable information for the GQDs application in biomedical field.

## Methods

### Synthesis of graphene quantum dots

NH_2_-GQDs (aGQDs) were prepared according to a previous study reported by Jiang et al. [6]. GO stock solution (2.5 mL of 4 mg/mL) was added to a vigorously stirred mixture of 5 mL of ammonia (25% to 28%) and 20 mL of H_2_O_2_ (30%). The gray turbid solution was heated to 80°C in a 50-mL conical flask. About 30 min later, the mixed solution became clear and the reaction continued for 24 h. The unreacted H_2_O_2_ and ammonia were removed by vacuum drying at 45°C. Finally, the product was dissolved with double-distilled water.

COOH-GQDs (cGQDs) were gained by pyrolyzing 2 g of citric acid at 200°C in a 5-mL beaker [9]. About 30 min later, the liquid became orange, implying the formation of cGQDs. The obtained orange liquid was added to 100 mL of 10 mg/mL^−1^ NaOH solution drop by drop under vigorous stirring. When the pH was adjusted to 7 with HCl, the resulting yellow-green liquid was dialyzed for 48 h in a 3,500 Da dialysis bag to obtain pure cGQDs.

CO-N (CH_3_)_2_-GQDs (dGQDs) were prepared with a top-down method using GO, H_2_O_2_, and N,N-dimethylformamide (DMF) as starting materials. The preparing process was similar to that of aGQDs except replacing ammonia with DMF. The unreacted H_2_O_2_ and water were removed by vacuum drying, and the residual DMF was removed through dialyzing for 48 h in a 3,500-Da dialysis bag.

### Characterization of GQDs

The UV-visible (vis) spectra and fluorescence spectra were obtained using a UV–Vis spectrometer (NanoDrop, Wilmington, DE, USA) and a fluorescence spectrometer (PerkinElmer, Waltham, MA, USA), respectively. Transmission electron microscopy (TEM) observation was performed on a JEM-2100HR transmission electron microscopy (JEOL, Akishima-shi, Japan) operated at 200 kV. Fourier transform infrared (FTIR) spectra were collected using a Tensor 27 FTIR spectrometer (Bruker, Karlsruhe, Germany) in the range 400 to 4,000 cm^−1^.

### Cell culture

A549 and C6 cells were cultured in Dulbecco’s modified Eagle medium (DMEM) supplemented with 10% (*v/v*) fetal bovine serum (FBS), penicillin (100 units/mL), and streptomycin (100 μg/mL) at 37°C in an incubator with 5% CO_2_ and 95% air.

### Cell imaging

After incubated with GQDs (50 μg/mL) for 12 h, cells adhered on coverslips were washed thoroughly with PBS three times. Formaldehyde (4%) was added to fix the cells for 20 min at room temperature. The cells without GQDs were taken as control. The cell imaging and distribution experiment was conducted by a fluorescence microscope (Leica, Wetzlar, Germany).

### MTT assay

The cytotoxicity of three modified GQDs was quantitatively evaluated by thiazoyl blue colorimetric (MTT) assay. Cells seeded in 96-well plates were separately treated with different concentrations (0, 10, 25, 50, 100, and 200 μg/mL) of aGQDs, cGQDs, and GQDs for 24 h. Ten microliters of MTT (5 mg/mL) was added to each well and incubated for another 4 h at 37°C. Next, 100 μL DMSO was added to each well, and the optical density at 490 nm was recorded on a microplate reader (Rayto, Shenzhen, China).

### Trypan blue assay

Cells were seeded in 6-well plates and incubated for 24 h. GQDs modified with different functional groups were separately introduced into cells with different concentrations (0, 10, 25, 50, 100, and 200 μg/mL). The cells in the supernatant and the adherent cells were collected and washed with PBS twice after incubation with GQDs for 24 h. Next, the cells were stained with 0.04% trypan blue solution for 3 min. The live and dead cells were counted using a cytometer.

### Flow cytometry experiment

Flow cytometry analysis was performed to detect apoptotic and necrotic cells on a FACSCanto™ flow cytometer (BD Biosciences, Heidelberg, Germany). Apoptosis or necrosis was analyzed by double staining with annexin V-fluorescein isothiocyanate (FITC) and propidium iodide (PI) according to the instructions of the manufacturer. The FITC positive control was prepared by culturing the control cells in medium containing 1% of H_2_O_2_ for 24 h. The PI positive control was designed by keeping the cells in a 70°C water bath for 20 min.

### Raman experiment

Raman spectra of cells were collected using a Renishaw inVia microspectrometer equipped with a semiconductor laser (785 nm) and a Leica DM2500 microscope (Leica). A × 50 objective was used to focus the laser beam and to collect the Raman signal. The Raman spectra were recorded in the range of 600 to 1,700 cm^−1^. Before the cell Raman spectra was obtained, the Raman band of silicon wafer at 520 cm^−1^ was obtained to calibrate the spectrometer and all the data were collected under the same conditions. All experiments were independently carried out at least five times. All the Raman spectra were baseline-corrected, removing the fluorescence background using a Vancouver Raman Algorithm software [28].

### Statistical analysis

The data of MTT assay, trypan blue assay, and flow cytometry experiment were presented as mean and standard deviation. Independent sample *t* test was used to analyze the differences between the treated groups and the control groups, and *p* value less than 0.05 was considered statistically significant.

## Results and discussion

### Synthesis and characterization of GQDs

**Figure 1:**
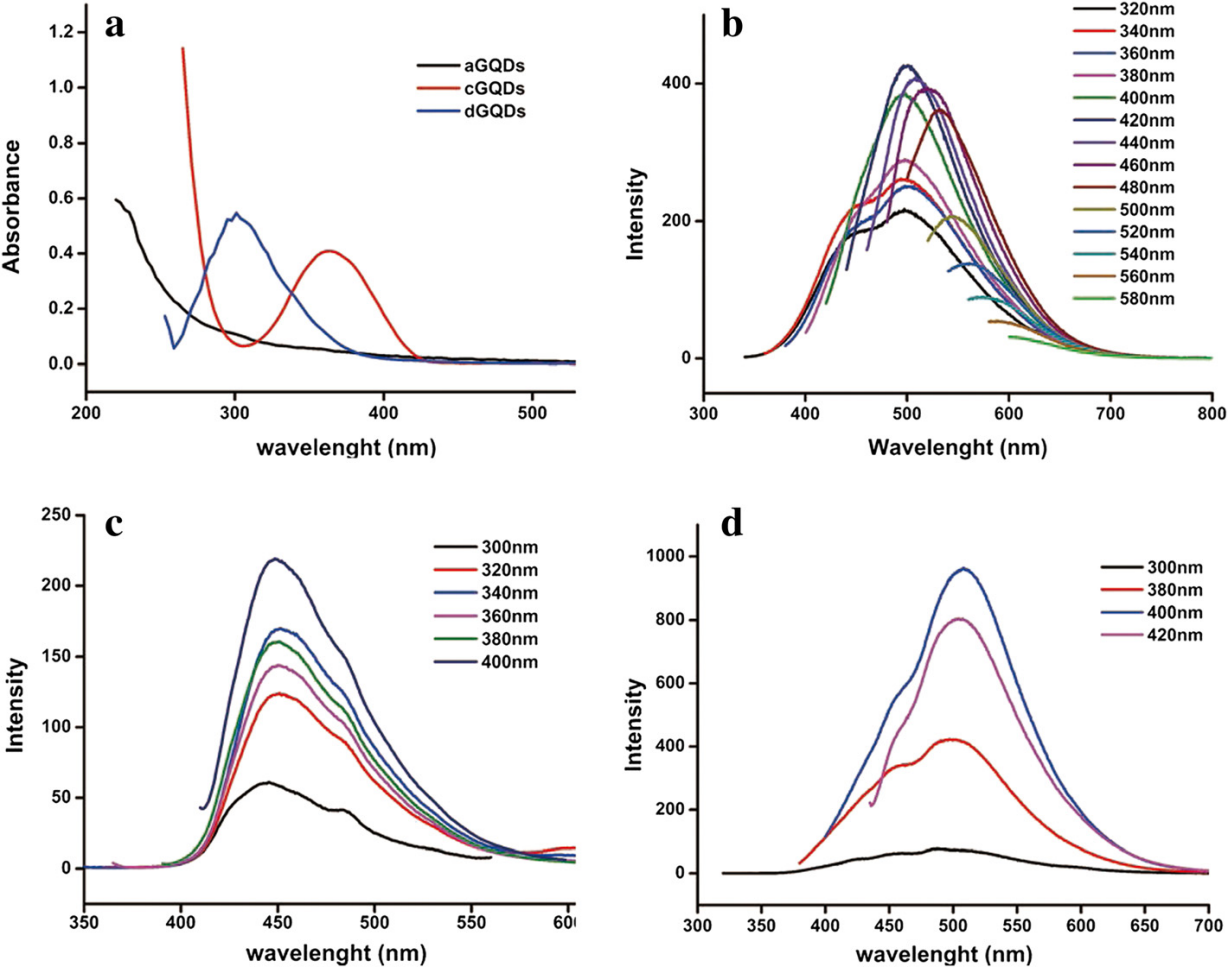
**UV–Vis absorption spectra and fluorescence spectra of three kinds of GQDs. (a)** The UV–Vis absorption spectra of three kinds of GQDs. **(b)** The fluorescence spectra of aGQDs excited from 320 to 580 nm. **(c)** The fluorescence spectra of cGQDs independent on the excitation wavelength. **(d)** The fluorescence spectra of dGQDs.

Figure 1a displayed the UV–Vis spectra of the three GQDs. The UV–Vis absorption spectra of aGQDs showed characteristic peak at around 230 nm and the absorption intensity decreased with the increasing wavelength, which was consistent with the previous report [6]. The characteristic absorption peak of cGQDs was at 362 nm with a narrow full width at half maximum of 60 nm, which was similar to previous reports [6,9]. Whereas, the UV–Vis analysis revealed that the absorption of dGQDs was at 300 nm, and the full width at half maximum was 56 nm.

As shown in Figure 1b, the fluorescence emission of aGQDs was excitation-dependent. The emission peaks shifted from 470 to 600 nm when the excitation wavelength was changed from 320 to 580 nm in a 20-nm increment. The strongest fluorescence peak was at 500 nm with 420 nm as the excitation wavelength, which was in agreement with a previous report [6]. Whereas, the emission peak of cGQDs and dGQDs were excitation-independent (Figure 1c,d). The maximum excitation wavelength and the maximum emission wavelength were at 400 and 440 nm for cGQDs and 400 and 500 nm for dGQDs, respectively.

**Figure 2:**
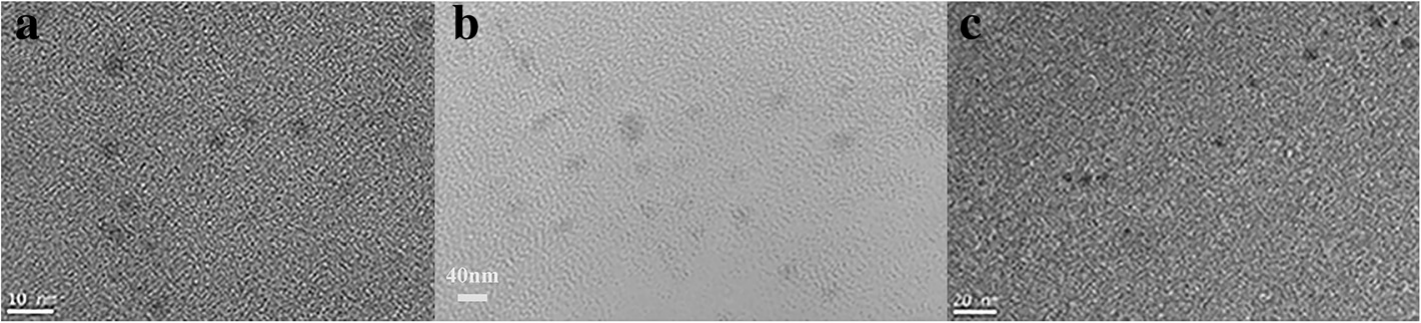
**TEM images of three modified GQDs deposited on copper grids. (a)** The TEM image of aGQDs. **(b)** Diameter distribution of the cGQDs. **(c)** The TEM image of dGQDs.

As can be seen in Figure 2, TEM images indicated that the average size of aGQDs was about 7.5 nm (Figure 2a) and the cGQDs was about 15 nm and they were monodispersed (Figure 2b), which were in accordance with previous reports [6,9]. The diameters of dGQDs mainly ranged from 3 to 10 nm (7.5 nm average diameter), and they were also monodispersed (Figure 2c). The monodispersed property of the dGQDs could be attributed to the rapid, vigorous stirring and the cracking effect of H_2_O_2_.

**Figure 3:**
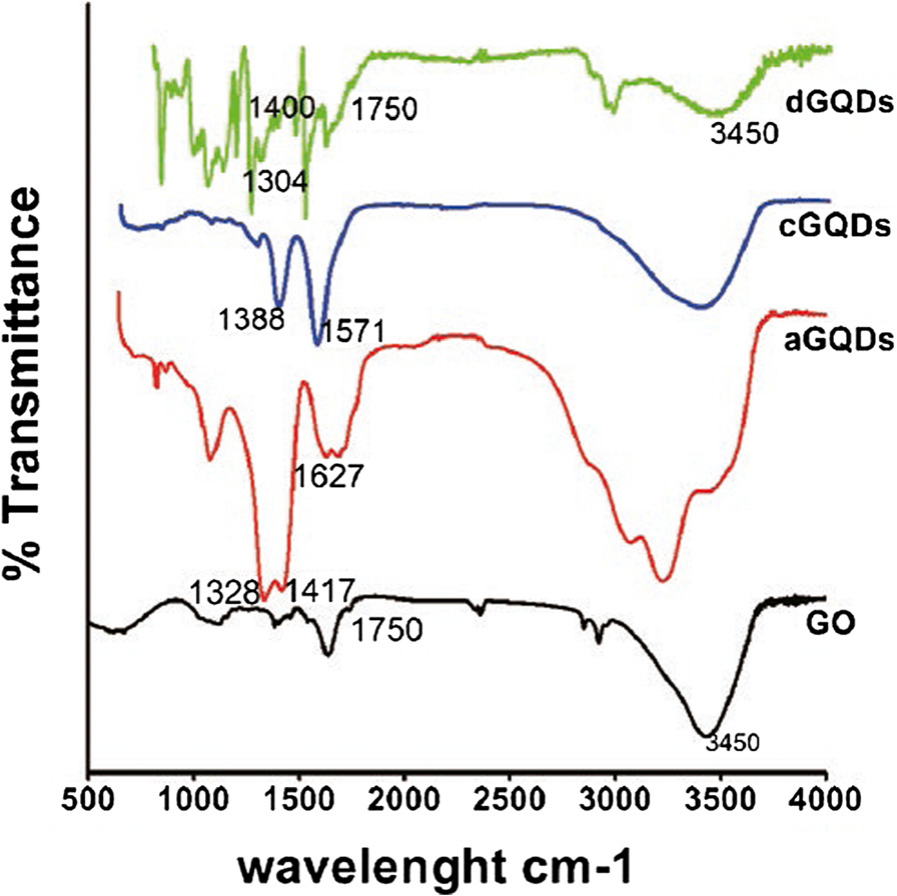
**FTIR spectra of the GQDs.** The FTIR spectra of three modified GQDs and GO.

As shown in Figure 3, in the aGQDs FTIR spectra, the peak at 1,627 cm^−1^ was attributed to the vibration of C = O bonds. The peak centered at 1,417 cm^−1^ was assigned to the bending vibrations of N-H bonds, while the peak at 1,328 cm^−1^ was attributed to the bending vibrations of C-N bonds, indicating that the amide functional groups had been successfully grafted onto the graphitic sheet. The FTIR spectra of cGQDs showed absorption of carboxyl group and hydroxyl group, as evidenced by the COO^−^ symmetric stretching vibration at 1,388 cm^−1^ and the COO^−^ antisymmetric stretching vibration at 1,571 cm^−1^[6,9]. In comparison with GO, two new peaks (1,400 and 1,304 cm^−1^) ascribed to the stretching vibration of C-N band emerged in the FTIR spectra of dGQDs, which implied that the CO-N (CH_3_)_2_ groups had been incorporated in the GQDs.

### The cell uptake and distribution of GQDs

**Figure 4:**
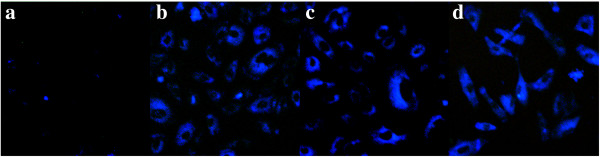
**Representative fluorescence microscope images of cells. (a)** Fluorescence image describing control cells. **(b)** Cells treated with 50 μg/mL of aGQDs for 12 h. **(c)** Cells exposed to 50 μg/mL of cGQDs for 12 h. **(d)** Cells after the treatment of 50 μg/mL of dGQDs for 12 h. Magnification, ×20.

The photoluminescent properties of the GQDs allow us to monitor their cellular uptake and distribution directly. GQDs uptake and bioimaging experiments were performed with a fluorescence microscope. In comparison with the control cells (Figure 4a) without GQDs that had been incubated for the same time, the fluorescence of the cells incubated with 50 μg/mL of modified GQDs (Figure 4b,c,d) for 12 h was obviously brighter, which indicated the cell uptake of GQDs with different chemical groups. The majority of the fluorescence intensity was raised from the cytoplasm, demonstrating that the three modified GQDs were located in the cytoplasm but not in the nucleus. No obvious reduction in fluorescence brightness was observed under continuous excitation over 20 min, indicating the high photostability of three kinds of modified GQDs.

### Cell proliferation evaluation

**Figure 5:**
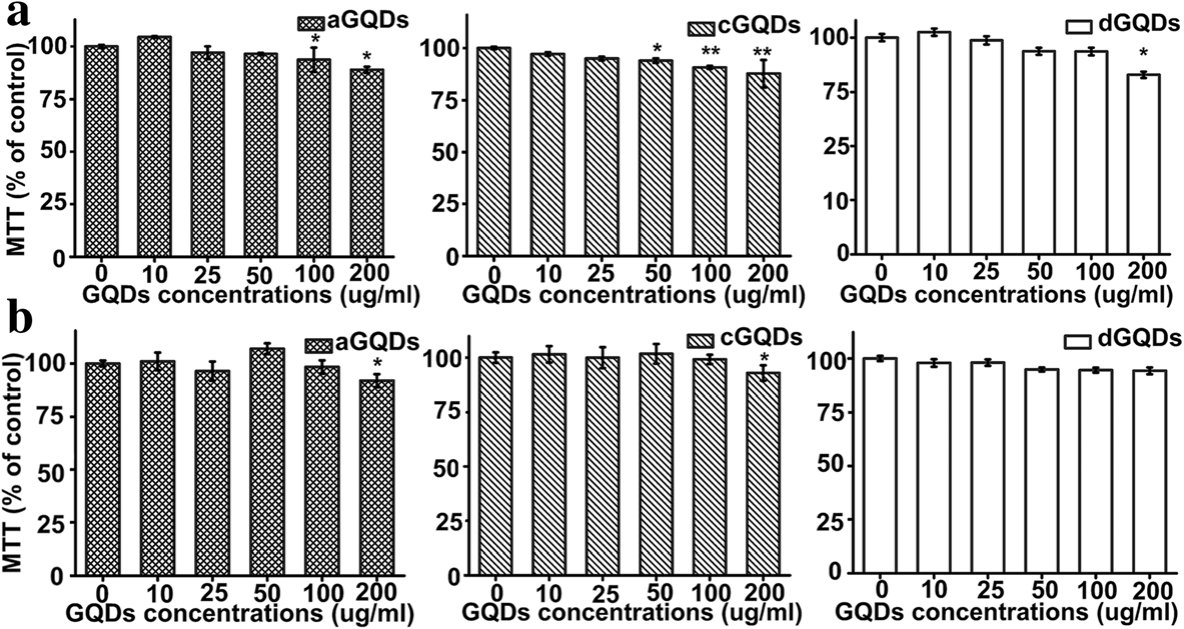
**The MTT (% of control) evaluated after exposed to three kinds of GQDs for 24 h. (a)** MTT (% of control) of A549 cells after exposed to different concentrations of three kinds of GQDs. **(b)** MTT (% of control) of C6 after the exposure to three kinds of GQDs at different concentrations. Asterisk indicated *p* < 0.05 and double asterisk represented *p* < 0.01.

Figure 5a showed that after 24-h exposure to aGQDs, the cell proliferation of A549 cells exhibited a concentration-dependent decrease. A significant cell proliferation decrease was induced by aGQDs when the concentration reached 100 and 200 μg/mL compared to that of the control cells (*p* < 0.05). When the concentration of cGQDs reached 50 μg/mL, the cell MTT (% of control) was statistically different from the control groups (*p* < 0.05). The influence of dGQDs on A549 cell proliferation was statistically significant only when the concentration was 200 μg/mL (*p* < 0.05). In brief, the influence of three kinds of modified GQDs on cell MTT (% of control) existed because the growth and proliferation of the cells slowed down when the GQDs concentrations were high. However, even at the highest concentration of 200 μg/mL, more than 80% of the cell MTT (% of control) still remained, implying that GQDs with different functional groups possessed good compatibility and low cytotoxicity. The results indicated that different chemical modifications made little difference on the cytotoxicity of GQDs. As far as we know, many studies have shown that GO had higher cytotoxicity than GQDs [29-31]. For instance, Zhang et al. reported that the GO had obvious cytotoxicity to HeLa cells even at low concentrations [29]. The results from previous studies reported by Wang et al*.* showed that GO possessed higher toxicity than GQDs [30]. The reason why GQDs exhibited more biocompatibility than GO might be that they are smaller and led to less damage to cell membrane. The good biocompatibility of the three modified GQDs was not cell specific, which was evidenced by the similar results gained from the C6 cells as shown in Figure 5b.

### Cell mortality analysis

**Figure 6:**
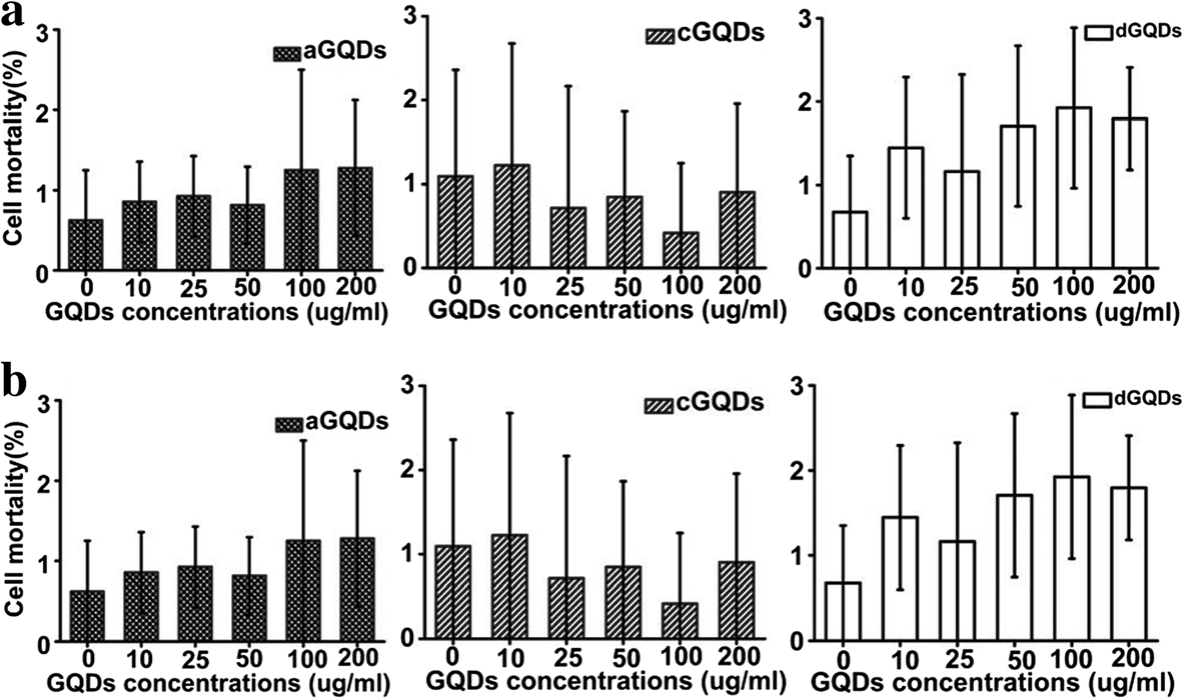
**The influence of GQDs with different functional groups on the mortality of cells. (a)** Cell mortality of A549 cells after treated with different concentrations of three GQDs. **(b)** Cell mortality after exposed to different concentrations of three kinds of GQDs evaluated in C6 cell line. Asterisk indicated *p* < 0.05 and double asterisk represented *p* < 0.01.

To provide a more comprehensive assessment of the cytotoxicity of GQDs with different functional groups, trypan blue assay was carried out to investigate the cell mortality induced by the three GQDs. No obvious mortality increase was observed after treated with the three GQDs even at the concentration of 200 μg/mL. As can be seen in Figure 6a, the cell mortality constantly remained below 2% after the exposure to different concentrations of aGQDs, cGQDs and dGQDs for 24 h. No significant differences between the GQDs treated cells and the control cells (about 1%) were observed in the mortality. Similar results acquired from C6 cells, as can be seen in Figure 6b, demonstrated that the biocompatibility and low cytotoxicity of the three GQDs with different functional groups were cell nonspecific.

### Flow cytometric analysis of apoptosis or necrosis

**Figure 7:**
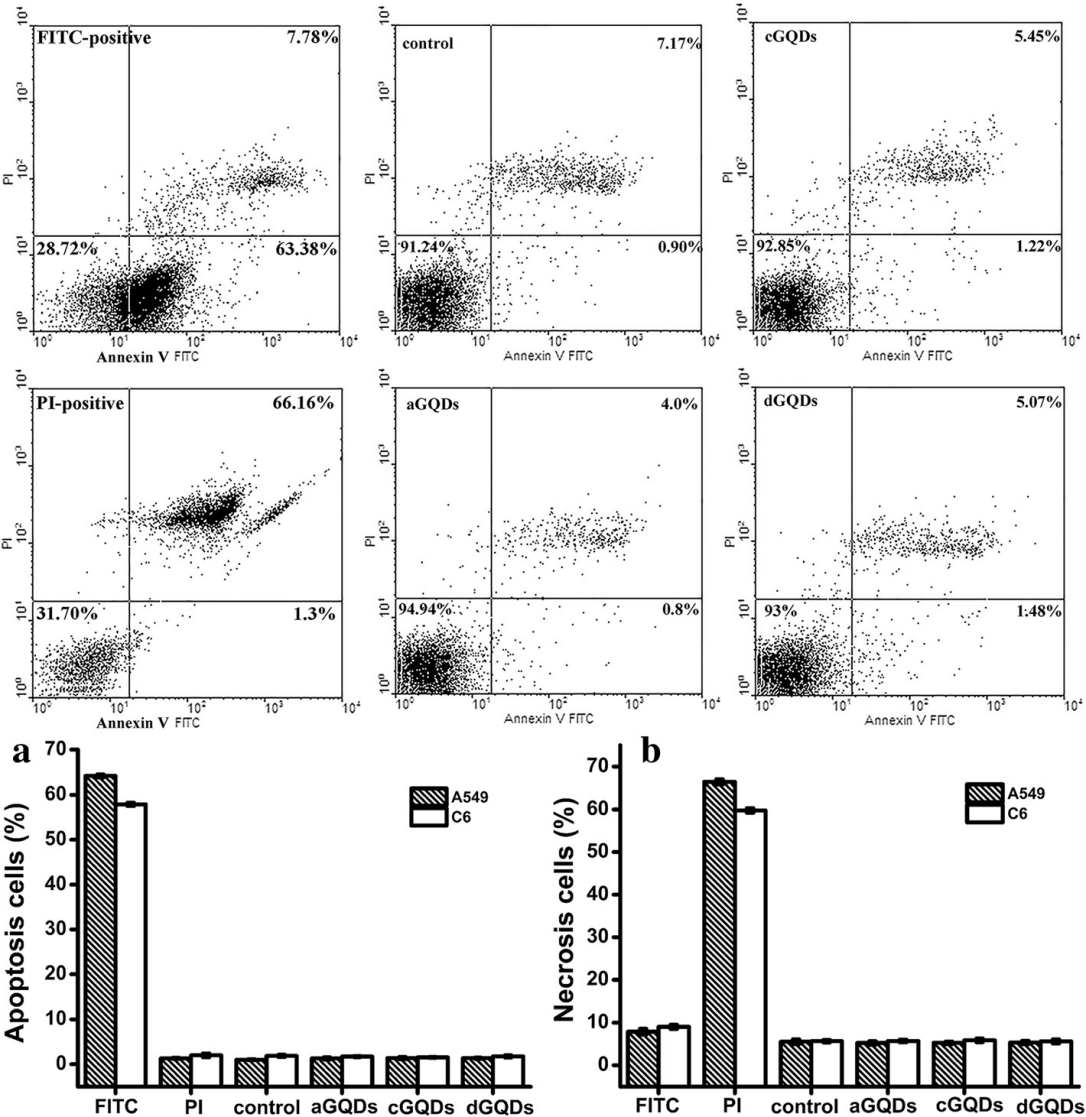
**Representative FACS images and the statistical results of cell apoptosis rate and necrosis rate.** After exposed to 200 μg/mL of the three kinds of GQDs. **(a)** Statistical results of cell necrosis. **(b)** Statistical results of cell apoptosis.

The type of cell death after exposed to the three kinds of GQDs was analyzed by double staining with annexin V-FITC and PI. Figure 7 showed the representative fluorescence-activated cell sorting (FACS) images and the statistical results of apoptosis and necrosis rate assessed by FACS analysis. The three kinds of GQDs did not induce obvious cell apoptosis or necrosis; more than 90% of cells were in the third quadrant even when the concentration was 200 μg/mL. The statistical data demonstrated that even when the GQDs concentration was at 200 μg/mL, the apoptosis rate (1.0% to 1.5%) and necrosis rate (5.5% to 5.8%) were still comparative with that of the control cells (1.1% and 5.6%, respectively).

### Raman spectral analysis

**Figure 8:**
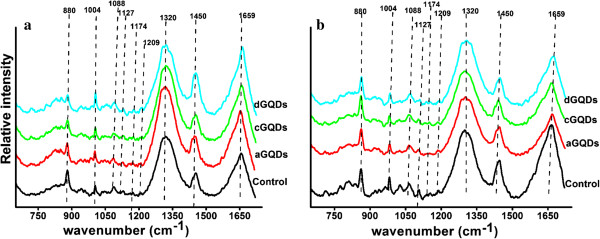
**Raman spectra of cells. (a)** Mean Raman spectra of A549 cells before and after exposure to 200 μg/mL of GQDs. **(b)** Average Raman spectra of C6 cells before and after treated with GQDs at the concentration of 200 μg/mL. Excitation wavelength, 785 nm.

To further investigate the influence of the three modified GQDs on the cells, the Raman spectra of cells were explored. Based on inelastic light scattering, Raman spectroscopy measures molecular vibrations and provides ‘fingerprint’ signatures of cell components, such as proteins, lipids, and nucleic acids [32,33]. Figure 8 depicted the average Raman spectra of cells, where ‘a’ was for A549 cells and ‘b’ was for C6 cells. Nine main bonds were observed in the Raman spectra: C-C symmetric stretching in lipids (880 cm^−1^), phenylalanine (1,003 cm^−1^), C-N stretching in proteins (1,088 cm^−1^), C-N, C-C stretching in proteins (1,127 cm^−1^), tyrosine and phenylalanine (1,174 cm^−1^), C-C_6_H_5_ stretching of phenylalanine (1,209 cm^−1^), CH deformation in proteins (1,320 cm^−1^), CH deformation in DNA/RNA, proteins, lipids, and carbohydrates (1,450 cm^−1^), and amide I α-helix (1,659 cm^−1^) [34-37]. In comparison with the control cells, no obvious changes in Raman shift and Raman intensity were observed in the spectra of cells treated with the GQDs even at the concentration up to 200 μg/mL. The results provided molecular level evidence for the biocompatibility and low cytotoxicity of aGQDs, cGQDs, and dGQDs.

## Conclusions

The present study investigated the cell distribution of three GQDs modified with different functional groups and compared their cytotoxicity in A549 and C6 cells. The fluorescent images of cells indicated that the GQDs accumulated in the cytoplasm but not in the nucleus after incubation for 12 h. When the concentration reached 50 μg/mL, three GQDs can illuminate the cells effectively. It was demonstrated that the three GQDs induced slight cell proliferation decreases at high concentrations. However, no visible mortality and apoptosis or necrosis increases resulted from the treatment of the three GQDs even at the concentration of 200 μg/mL. In addition, the Raman spectroscopy experiment provided molecular level evidence for the biocompatibility of the three kinds of GQDs, revealing that no obvious changes in cell Raman spectra were generated by the treatment of 200 μg/mL GQDs. In summary, the results manifested that when modified with different chemical groups, GQDs still possessed excellent biocompatibility and low cytotoxicity to cells, which may make them more promising in bioimaging and other biomedical applications.

## Abbreviations

aGQDs: NH_2_-graphene quantum dots; cGQDs: COOH-graphene quantum dots; dGQDs: CO-N (CH_3_)_2_-graphene quantum dots; DMEM: Dulbecco’s modified Eagle medium; FITC: V-fluorescein isothiocyanate; GQDs: Graphene quantum dots; GO: graphene oxide; MTT: thiazoyl blue colorimetric; PI: propidium iodide; QY: quantum yield.

## Competing interests

The authors declare that they have no competing interests.

## Authors’ contributions

XY, ZL, and YJ conceived and designed the study. XY, ZL, and MJ carried out the experiments and analyzed the data. XY wrote the paper, and ZL, ZG, and XW corrected the paper. All authors read and approved the final manuscript.

## Authors’ information

XY, MJ, and XW are master’s degree candidates. ZL is a researcher assistant, and YJ is an associate researcher. ZG is a deputy director and professor.
